# Loss of UFL1 confers enzalutamide resistance of prostate tumors by regulating METTL16-mediated m6A modification of EEF1A1 mRNA

**DOI:** 10.7150/ijbs.124214

**Published:** 2026-01-01

**Authors:** Xu Wu, Hui Gao, Qianfeng Zhuang, Feng Li, Xingliang Feng, Naiyuan Shao, Wei Zhang, Yuyang Zhang, Xiansheng Zhang

**Affiliations:** 1 Department of Urology, The First Affiliated Hospital of Anhui Medical University, Hefei 230022, China.; 2 Anhui Province Key Laboratory of Urological and Andrological Diseases Research and Medical Transformation, Anhui Medical University, Hefei 230022, China.; 3 Department of Urology, The Third Affiliated Hospital of Soochow University, Changzhou 213003, Jiangsu, China.; 4 Department of Neurosurgery, The Third Affiliated Hospital of Soochow University, Changzhou, Jiangsu, China.; 5 Department of Urology, The Second Affiliated Hospital of Anhui Medical University, Hefei 230601, China.; 6 Reproductive Medicine Center, Department of Obstetrics and Gynecology, the First Affiliated Hospital of Anhui Medical University, Hefei 230022, China.

**Keywords:** UFL1, enzalutamide resistance, METTL16, EEF1A1

## Abstract

Enzalutamide (ENZ), a next-generation androgen receptor (AR) inhibitor, is a cornerstone treatment for metastatic prostate cancer. However, resistance to ENZ inevitably develops in these patients, and the mechanisms underlying this resistance remain poorly understood. This study reveals that UFL1 is dysregulated in ENZ-resistant cells, xenograft models, and prostate tumors. UFL1 deficiency enhances prostate cancer cell resistance to ENZ both *in vitro* and *in vivo*. Mechanistically, UFL1 loss decreases METTL16 UFMylation, thereby reducing its ubiquitination level and increasing its protein stability. Additionally, METTL16-mediated m6A modification of EEF1A1 mRNA activates the m6A-IGF2BP1 axis, resulting in increased EEF1A1 protein levels and enhanced resistance to ENZ-induced apoptosis. These findings uncover a novel UFL1-METTL16-EEF1A1 signaling pathway that drives ENZ resistance, suggesting that targeting this cascade may offer a promising therapeutic strategy for overcoming ENZ resistance in prostate cancer.

## Introduction

Prostate cancer (PCa) emerges as the most prevalent malignant tumor impacting male populations and ranks second in cancer-related mortality globally^1^. Considering the essential function of androgen receptor (AR) signaling in PCa development, androgen deprivation therapy (ADT) has conventionally served as the predominant therapeutic strategy for individuals presenting with localized advanced or metastatic PCa. Nonetheless, nearly all patients inevitably transition to castration-resistant prostate cancer (CRPC), a lethal phase lacking curative therapeutic interventions^2^, ^3^. Enzalutamide (ENZ), a second-generation AR pathway inhibitor, attaches to the AR's ligand-binding domain, blocking its nuclear translocation and suppressing AR-mediated transcriptional processes^4^-^6^. Although ENZ extends survival in patients with CRPC, resistance invariably develops, and therapeutic options post-resistance are limited^7^, ^8^. This emphasizes the critical necessity for innovative therapeutic targets to address enzalutamide resistance (ENZR).

The ubiquitin-like protein Ubiquitin-fold modifier 1 (UFM1) establishes covalent linkages with lysine residues through a sequential enzymatic process encompassing UBA5 (an E1-like activating enzyme), UFC1 (an E2-like conjugating enzyme), and UFL1 (the exclusive recognized E3-like ligase for UFM1), which closely parallels the conventional ubiquitination pathway^9^. UFMylation is a dynamic and reversible post-translational modification, with UFM1-specific proteases (UFSPs) catalyzing the removal of UFM1 from modified proteins^10^. Two UFSP genes are encoded in humans: UFSP1, which processes UFM1 precursors, and UFSP2, which predominantly mediates deconjugation of UFM1 from target proteins. Despite UFMylation's critical roles in embryonic development, endoplasmic reticulum homeostasis, and DNA damage repair^9^-^17^, the full range of UFMylation substrates remains limited, and its role in PCa and ENZR is poorly understood.

Epitranscriptomic RNA modifications, especially N6-methyladenosine (m6A)—recognized as the primary internal modification within eukaryotic mRNA—have emerged as key regulators of tumorigenesis and therapeutic resistance^18^, ^19^. Dysregulated expression of m6A regulatory proteins has been implicated in various cancers, contributing to malignant progression and drug resistance^20^-^22^. METTL16, in addition to the canonical METTL3/METTL14 complex, was recently identified as a second m6A “writer.” METTL16 mediates m6A deposition on multiple transcripts, including MAT2A (encoding S-adenosylmethionine synthetase) and U6 snRNA, and promotes tumorigenesis in an m6A-dependent manner across several malignancies^23^-^26^. Nevertheless, the function of METTL16 in PCa and ENZR remains largely unexplored.

This investigation characterized METTL16 as a previously unidentified substrate of UFMylation. Loss of UFL1 decreases METTL16 UFMylation, resulting in its stabilization by preventing ubiquitin-mediated proteasomal degradation. Functionally, METTL16 promotes ENZR in PCa by enhancing EEF1A1 expression in an m6A-dependent manner, thereby inhibiting ENZ-induced apoptosis. The investigation reveals a novel UFL1-METTL16-EEF1A1 pathway instrumental in ENZR progression, subsequently highlighting UFL1 as a potential strategic intervention point for addressing ENZR in CRPC.

## Results

### UFL1 expression is downregulated in ENZR PCa

**Figure 1:**
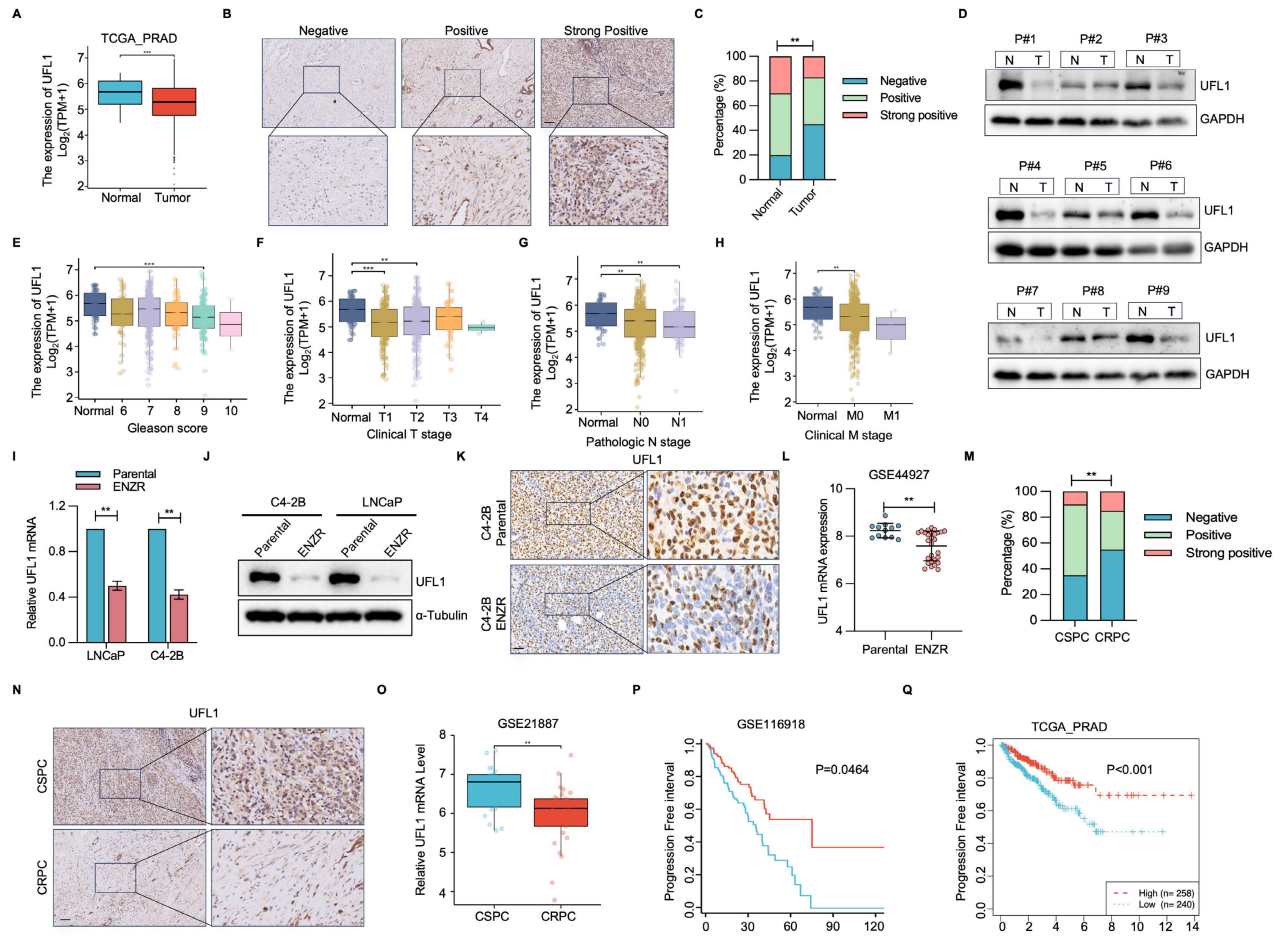
** UFL1 is downregulated in ENZR CRPC.** (A) Analysis of UFL1 level in human PCa samples from the TCGA-PRAD dataset. (B) Representative IHC staining images for UFL1 in PCa tissues. (C) Statistical analysis of UFL1 protein levels by IHC. (D) WB examination demonstrating UFL1 protein levels in fresh PCa tumors and paired normal tissues. (E-H) Correlation of UFL1 mRNA level with Gleason score (E), clinical stage (F), lymph node metastasis (G), and distant metastasis (H). (I-J) UFL1 levels in PCa cell lines. LNCaP-Parental, LNCaP-ENZR, C4-2B-Parental, and C4-2B-ENZR cells were procured and examined by qRT-PCR (I) and WB (J). (K) Representative IHC images of UFL1 staining in tumor sections from xenograft mice. (L) UFL1 expression in the public GSE44927 dataset. (M-N) Representative IHC images demonstrating UFL1 level in CSPC and CRPC tissues. (O) Examination of UFL1 expression in human PCa samples from GEO datasets. (P-Q) In the published PCa cohort (GSE116918) and TCGA-PRAD dataset, reduced UFL1 expression was correlated with worse overall survival. Scale bar, 50 µm. *P < 0.05, **P < 0.01, ***P < 0.001.

To elucidate the potential significance of UFL1 in PCa progression, UFL1 level was analyzed using patient datasets from the TCGA-PRAD, TNMplot, and GEO databases. Relative to normal prostatic tissues, UFL1 expression exhibited marked reduction in PCa samples (Figure 1A, Figure S1A, S1B). This finding was validated through assessment of UFL1 protein expression in a cohort comprising 10 primary and 20 PCa tissue samples (Figure 1B, 1C). Furthermore, 9 paired tumor and adjacent tissues from PCa patients underwent analysis, demonstrating markedly decreased UFL1 protein levels in 8/9 human PCa tissues relative to their paired adjacent tissues (Figure 1D). Diminished UFL1 expression exhibited a strong correlation with tumor size, lymph node metastasis, distant metastasis, clinical stage, and Gleason score (Figure 1E-H, Figure S1C).

To assess the correlation between UFL1 levels and ENZR *in vitro*, we established two pairs of enzalutamide-resistant PCa cell lines (LNCaP-ENR, C4-2B-ENR) (Figure S1D and S1E). qPCR and Western blot (WB) analyses showed a marked reduction in UFL1 level in ENZR (LNCaP-ENR, C4-2B-ENR) PCa cells (Figure 1I and 1J). Interestingly, the expression of DDRGK1 and UFC1, two key regulatory components of the UFMylation machinery, was also reduced in ENZR PCa cells (Figure S1F). Moreover, immunohistochemistry (IHC) confirmed that UFL1 protein expression was lower in ENZR xenografts compared to their parental tumors (Figure 1K). These observations aligned with UFL1 expression patterns observed in multiple additional ENZR cell lines derived from the GSE44927 dataset (Figure 1L). Consistent with these findings, IHC and GEO databases analysis validated that UFL1 protein levels are markedly diminished in CRPC relative to patients diagnosed with CSPC (Figure 1M-O and Figure S1E). Additionally, patients with low UFL1 expression exhibited worse disease-free survival in the GSE116918 and TCGA-PRAD datasets (Figure 1P, 1Q). These observations indicate that diminished UFL1 expression links to ENZR in PCa.

### UFL1 deficiency elevates the resistance of PCa cells to ENZ

**Figure 2:**
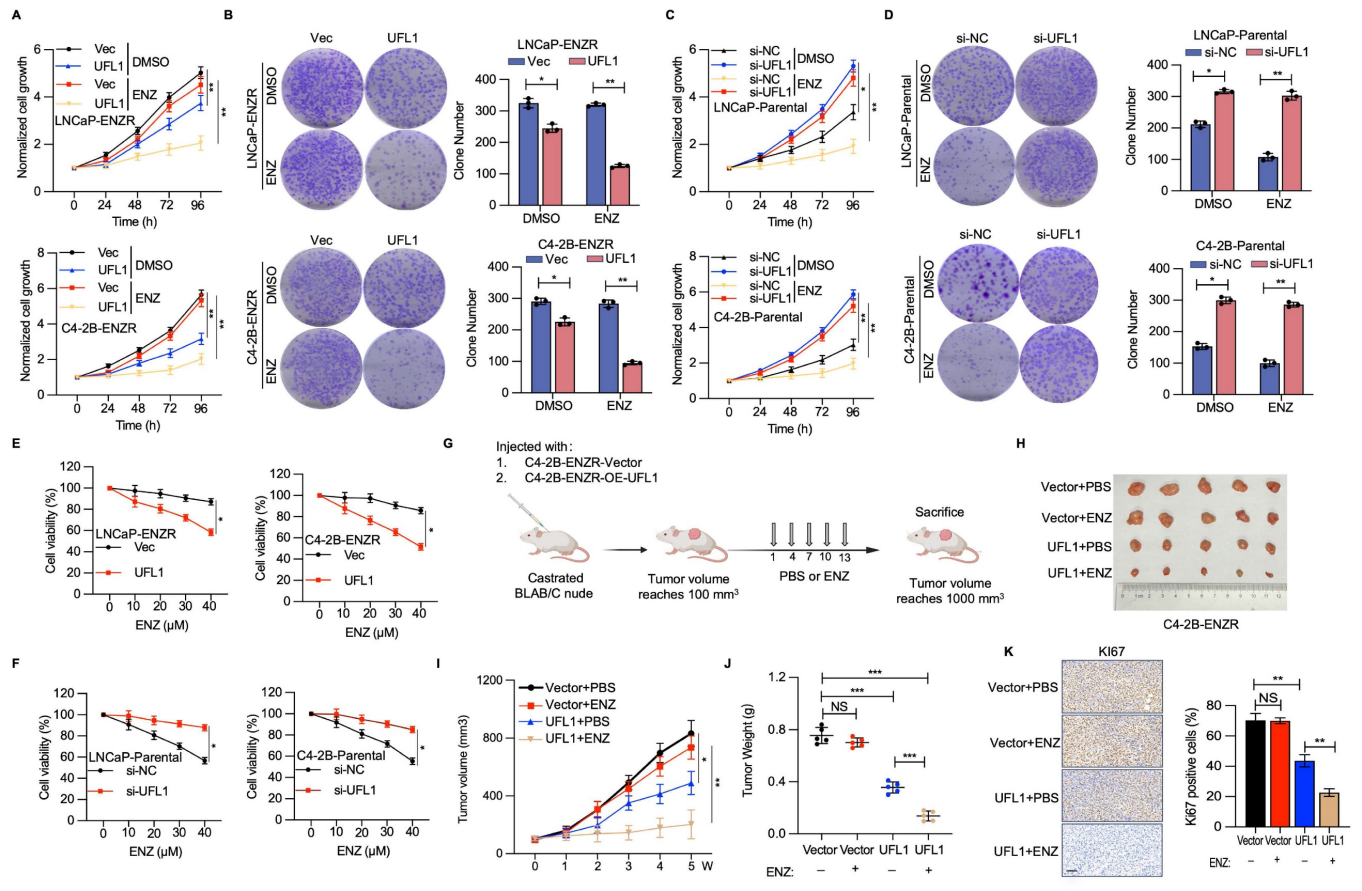
** Loss of UFL1 facilitates ENZR *in vitro* and *in vivo*.** (A-D) Cell proliferative capacity was evaluated through CCK-8 and colony formation assays in LNCaP-ENZR and C4-2B-ENZR cell lines following transfection with empty vector or UFL1 overexpression plasmid (A, B) and in LNCaP-Parental and C4-2B-Parental cell lines post-transfection with siNC or UFL1 siRNA (C, D). (E-F) Cell viability was evaluated in designated cell lines during ENZ treatment. LNCaP-ENZR, C4-2B-ENZR (E), and LNCaP-Parental, C4-2B-Parental (F) Cell lines were transfected as specified and exposed to graduated ENZ concentrations for 3 days. Cell viability was evaluated utilizing CCK-8 assays. (G) Experimental protocol for developing a xenograft mouse model with C4-2B-ENZR cells. (H-J) Impact of UFL1 overexpression in conjunction with ENZ treatment on tumor growth within a subcutaneous xenograft model. Tumor dimensions were assessed at designated time intervals, with tumors being harvested and weighed following mouse sacrifice. (K) Immunohistochemical staining images and quantification of Ki67 H-scores. ENZ, enzalutamide. Scale bar, 50 µm. *P < 0.05, **P < 0.01, ***P < 0.001.

To elucidate the potential implications of diminished UFL1 expression in PCa cells during ENZR progression, RNA interference methodologies were utilized to attenuate UFL1 expression in LNCaP-Parental and C4-2B-Parental cell lines, while implementing UFL1 overexpression strategies in LNCaP-ENZR and C4-2B-ENZR cells. UFL1 overexpression markedly decreased cell proliferation and colony formation in LNCaP-ENZR and C4-2B-ENZR cells (Figure 2A, 2B). Conversely, UFL1 depletion promoted cell proliferation and colony formation in LNCaP-Parental and C4-2B-Parental cells (Figure 2C, 2D). Furthermore, we found that elevated UFL1 expression diminished ENZ resistance in C4-2B-ENZR and LNCaP-ENZR cell lines, whereas suppressing UFL1 levels decreased ENZ susceptibility in C4-2B-Parental cells (Figure 2E, 2F).

To further investigate the influence of UFL1 on ENZR PCa cells, castrated mice received subcutaneous injections of C4-2B-ENZR cells with stable overexpression of UFL1 or control (Figure 2G and Figure S1H). When tumors achieved 100 mm³, all mice were randomly divided into two groups within each group. One group administered intraperitoneally with PBS, while the other group received ENZ, with ongoing monitoring of tumor growth. In alignment with *in vitro* observations, the elevated UFL1 level markedly strengthened ENZ's suppressive impact on C4-2B-ENZR tumor development, evidenced by a substantial decrease in both tumor dimensions and mass versus the control group (Figure 2H-J). IHC analysis of tumor tissues revealed that UFL1 overexpression led to less intense Ki-67 staining (Figure 2K). Moreover, Ki-67 protein expression was markedly decreased in UFL1-overexpressing tumors after ENZ treatment versus the control group (Figure 2K). These findings additionally validate the hypothesis that UFL1 deficiency contributes to enhanced resistance of PCa cells to ENZ.

### UFL1 deficiency stabilizes METTL16

**Figure 3:**
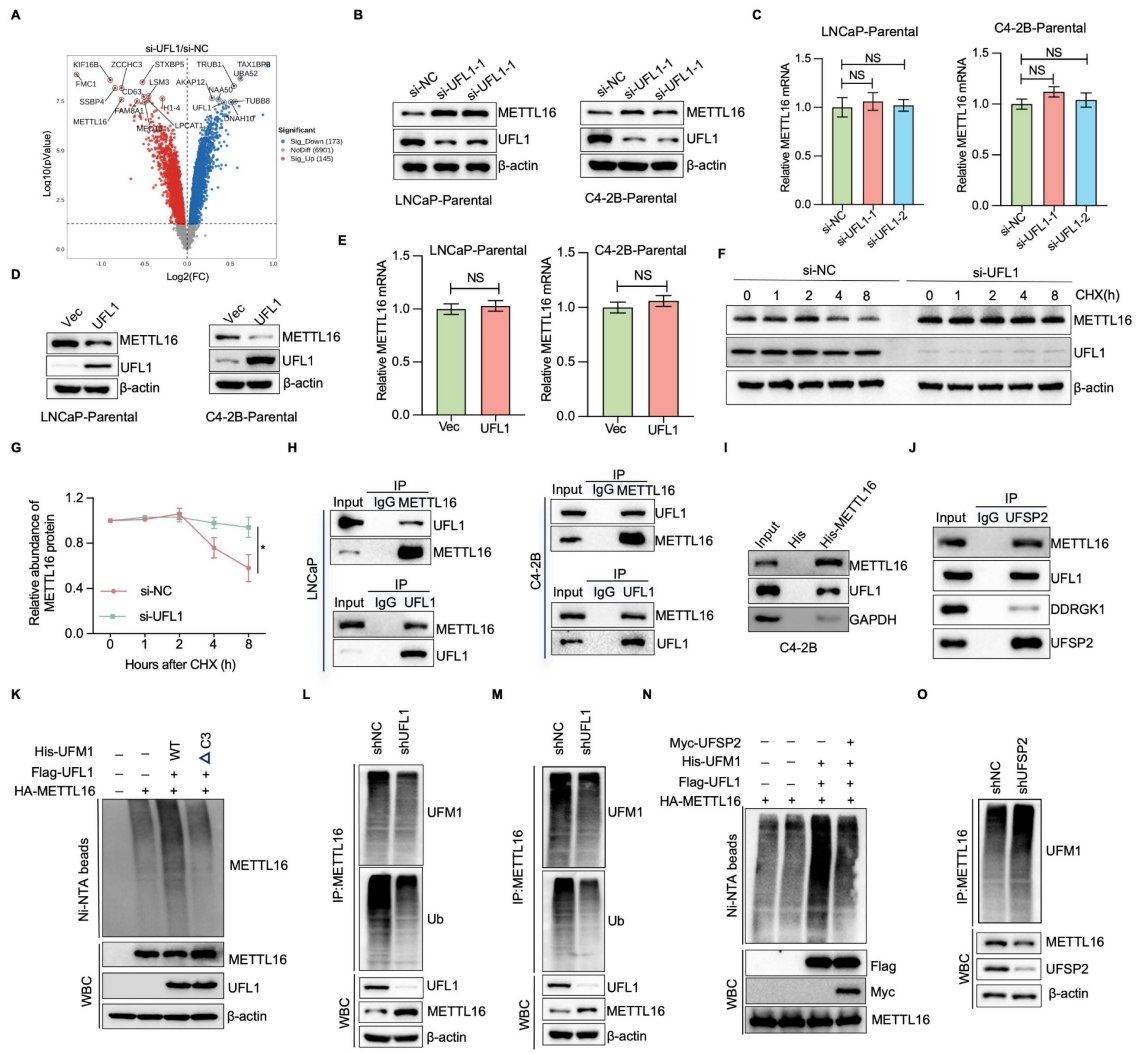
** UFL1 deficiency stabilizes METTL16.** (A) Volcano plot illustrating differentially expressed proteins derived from TMT-based proteomic analysis of C4-2B cells following transfection with si-NC or UFL1 siRNA. (B-C) WB analysis of whole cell lysates (WCL) obtained from si-NC or si-UFL1-treated C4-2B-Parental and LNCaP-Parental cells (B). METTL16 mRNA levels were examined using RT-qPCR (C). (D-E) WB analysis of WCL obtained from Vec or UFL1-treated C4-2B-ENZR and LNCaP-ENZR cells (D). METTL16 mRNA levels were examined using RT-qPCR (E). (F-G) METTL16 protein stability assessment through Western blot in UFL1 knockdown versus control C4-2B cells. Cells underwent cycloheximide (CHX) treatment at 100 μg/mL for specified durations. Protein level quantification of METTL16 is presented in (G). (H) Co-immunoprecipitation analysis exploring endogenous METTL16 and UFL1 interaction. (I) *In vitro* pull-down experiment utilizing purified 6*His-METTL16 and C4-2B cell lysate. (J) Protein interaction investigation among UFSP2, DDRGK1, UFL1, and METTL16 via co-immunoprecipitation. (K) UFMylation of ectopically expressed METTL16 assessed via Ni-NTA pull-down employing denaturing buffer A in HEK293T cells. (L-M) Endogenous METTL16 UFMylation examination via denatured-immunoprecipitation in shGFP/shUFL1-treated cells. (N) UFMylation of ectopically expressed METTL16 assessed through Ni-NTA pull-down in denaturing buffer A from HEK293T cells. (O) Endogenous METTL16 UFMylation investigation via immunoprecipitation in shNC/shUFSP2-treated C4-2B cells. *P < 0.05, **P < 0.01, ***P < 0.001.

Accumulating evidence suggests that, analogous to ubiquitin and additional ubiquitin-like protein modifications, UFMylation serves a crucial function in controlling substrate stability^9^-^11^, ^14^, ^15^. To investigate the mechanisms through which UFL1 promotes ENZR in PCa cells, we carried out TMT-based quantitative proteomics in C4-2B cells with or without UFL1 knockdown. The investigation demonstrated that 145 proteins exhibited elevated expression levels, while 173 proteins displayed decreased expression patterns in si-UFL1 cells versus si-NC cells (Figure 3A). Notably, METTL16 expression exhibited marked elevation in si-UFL1 cells versus si-NC cells. METTL16 has been documented to display aberrant overexpression across various malignancies, encompassing lung, cervical, squamous cell carcinoma, and chronic myelogenous leukemia, and serves a critical function in enhancing cell proliferation and invasiveness^27^, ^28^. Based on these observations, METTL16 was chosen for additional functional investigation in PCa. Verification studies showed that knockdown of UFL1 by two independent siRNAs the accumulation of METTL16 protein levels in LNCaP and C4-2B cells, while UFL1 overexpression decreased METTL16 protein levels (Figure 3B, 3D). Nevertheless, METTL16 mRNA levels showed no significant alteration between UFL1-deficient and proficient cells (Figure 3C, 3E), indicating that UFL1-mediated regulation of METTL16 occurs at the post-translational level. Furthermore, treatment with the proteasome inhibitor MG132 resulted in a marked elevation in METTL16 protein abundance in control cells, whereas UFL1-silenced cells failed to display such accumulation (Figure S1I), supporting the concept that UFL1 facilitates the proteasomal degradation of METTL16. Consistently, UFL1 knockdown markedly prolonged METTL16 protein half-life (Figure 3F, 3G).

To further investigate whether UFL1 interacts with METTL16 and promotes its UFMylation, co-immunoprecipitation assays were performed. As shown in Figure 3H, METTL16 were found to associate with UFL1 in both LNCaP and C4-2B cells. This interaction was substantiated through an *in vitro* pull-down assay, which demonstrated the binding of 6*His-METTL16 with C4-2B cell lysate (Figure 3I). Furthermore, METTL16 was observed to associate with additional UFMylation components, DDRGK1 and UFSP2 (Figure 3J), indicating that METTL16 serves as a genuine partner of UFL1. Considering that UFMylation represents a ubiquitin-like post-translational modification, METTL16 may function as a substrate for UFMylation. *In vivo* UFMylation assays revealed that UFL1 markedly elevated METTL16 UFMylation (Figure 3K). The C-terminally truncated UFM1 variant (UFM1^ΔC3^), lacking the final three amino acid residues (83Gly-Ser-Cys85), substantially impaired UFL1-dependent poly-UFM1 chain assembly targeting METTL16 (Figure 3K). Furthermore, METTL16 UFMylation was detectable at endogenous levels in both LNCaP and C4-2B cells (Figure 3L, 3M). Ectopic expression of UFSP2 reduced METTL16 UFMylation, whereas UFSP2 knockdown enhanced METTL16 UFMylation (Figure 3N, 3O). These findings indicate that METTL16 UFMylation undergoes regulation by essential elements of the UFMylation pathway, thereby affecting its stability.

### METTL16 is essential for PCa cell proliferation and ENZR

**Figure 4:**
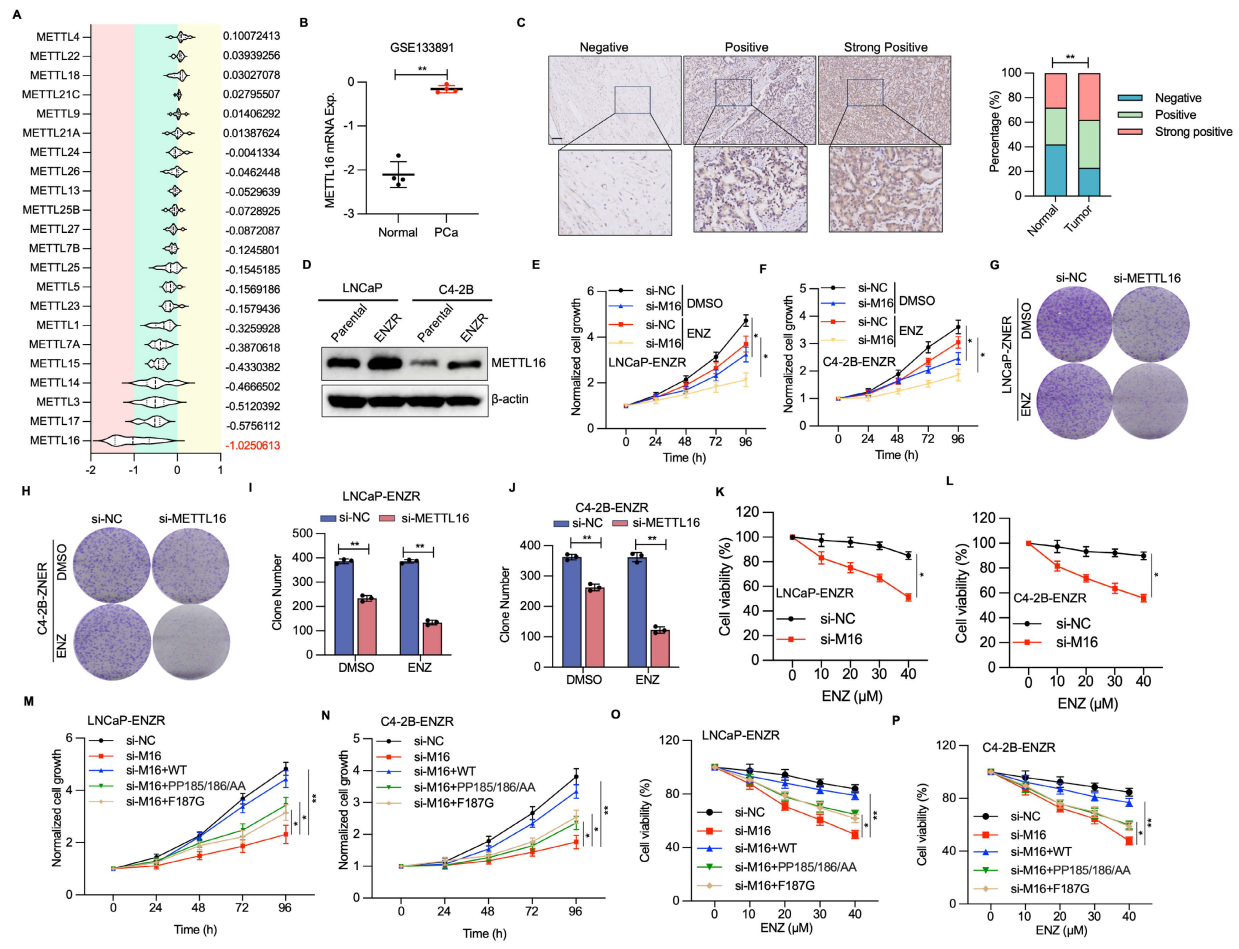
** METTL16 facilitates PCa cell growth, survival, and ENZR.** (A) Violin plots illustrating CERES scores for all METTL family members across 11 PCa cell lines. (B) Analysis of METTL16 expression in human PCa samples from GEO datasets. (C) Representative IHC staining images for METTL16 and a statistical graph of METTL16 protein levels. (D) METTL16 expression in PCa cell lines. LNCaP-Parental, LNCaP-ENZR, C4-2B-Parental, and C4-2B-ENZR cells were collected, and total lysates were analyzed by WB (D). (E-J) Cell proliferation assessment utilized CCK-8 assay and colony formation assay in LNCaP-ENZR and C4-2B-ENZR cells following transfection with siNC or METTL16 siRNA. (K-L) Cell viability determination occurred in the designated cell lines during ENZ treatment. LNCaP-ENZR and C4-2B-ENZR cells underwent transfection as specified and received graded ENZ doses over 3 days. Cell viability quantification employed CCK-8 assays. (M-N) Recovery effects of wild-type METTL16 and catalytically inactive METTL16 mutants on METTL16 knockdown-mediated cell proliferation inhibition in LNCaP-ENZR and C4-2B-ENZR cells. (O-P) Cell viability determination in specified cell lines under ENZ treatment. Scale bar, 50 µm. *P < 0.05, **P < 0.01, ***P < 0.001.

The function of METTL16 in PCa tumorigenesis underwent evaluation. Genome-wide CRISPR-Cas9 knockout screening data procured from DepMap across 11 PCa cell lines indicated that, within the entire METTL family^29^, METTL16 emerges as the most critical gene in PCa cellular contexts (Figure 4A). Furthermore, analysis of GEO datasets indicated that METTL16 expression exhibits upregulation in PCa tumors versus adjacent normal tissues (Figure 4B, Figure S2A). Consistent with these observations, immunohistochemical examination unveiled heightened METTL16 protein concentrations in prostate cancer tissue samples versus adjacent non-tumoral tissue regions (Figure 4C). Furthermore, elevated METTL16 levels demonstrated a marked link to diminished overall survival probability (Figure S2B).

To explore whether METTL16 levels correlate with ENZR, WB analysis demonstrated a notable elevation in METTL16 expression within ENZR (LNCaP-ENR, C4-2B-ENR) PCa cells (Figure 4D). Consistent with these findings, METTL16 expression was notably higher in CRPC compared to CSPC or primary PCa (Figure S2C, S2D). As anticipated, METTL16 depletion markedly diminished cell proliferation and colony formation in LNCaP-ENZR and C4-2B-ENZR cell lines (Figure 4E-J). Additionally, METTL16 suppression undermined the ENZ resistance of C4-2B-ENZR and LNCaP-ENR cells (Figure 4K, 4L).

To assess the role of METTL16's m6A methyltransferase activity in PCa cells, this study examined the effects of wild-type (WT) METTL16 and catalytically impaired mutants (PP185/186AA and F187G)^28^, ^30^. Introduction of METTL16-WT significantly promoted cellular proliferative capabilities, while catalytically impaired mutants (F187G and PP185/186AA) demonstrated markedly diminished cell proliferation and compromised cellular survival (Figure 4M, 4N). Furthermore, re-introduction of METTL16-WT rescued cell growth and ENZR in METTL16-depleted cells, whereas the catalytically-inactive METTL16 was unable to produce this effect (Figure 4O, 4P). These observations indicate that METTL16's methyltransferase functionality serves a pivotal function in driving PCa cell proliferation and ENZR.

### UFL1-METTl16 axis suppresses ENZ-induced apoptosis of ENZR PCa cells

**Figure 5:**
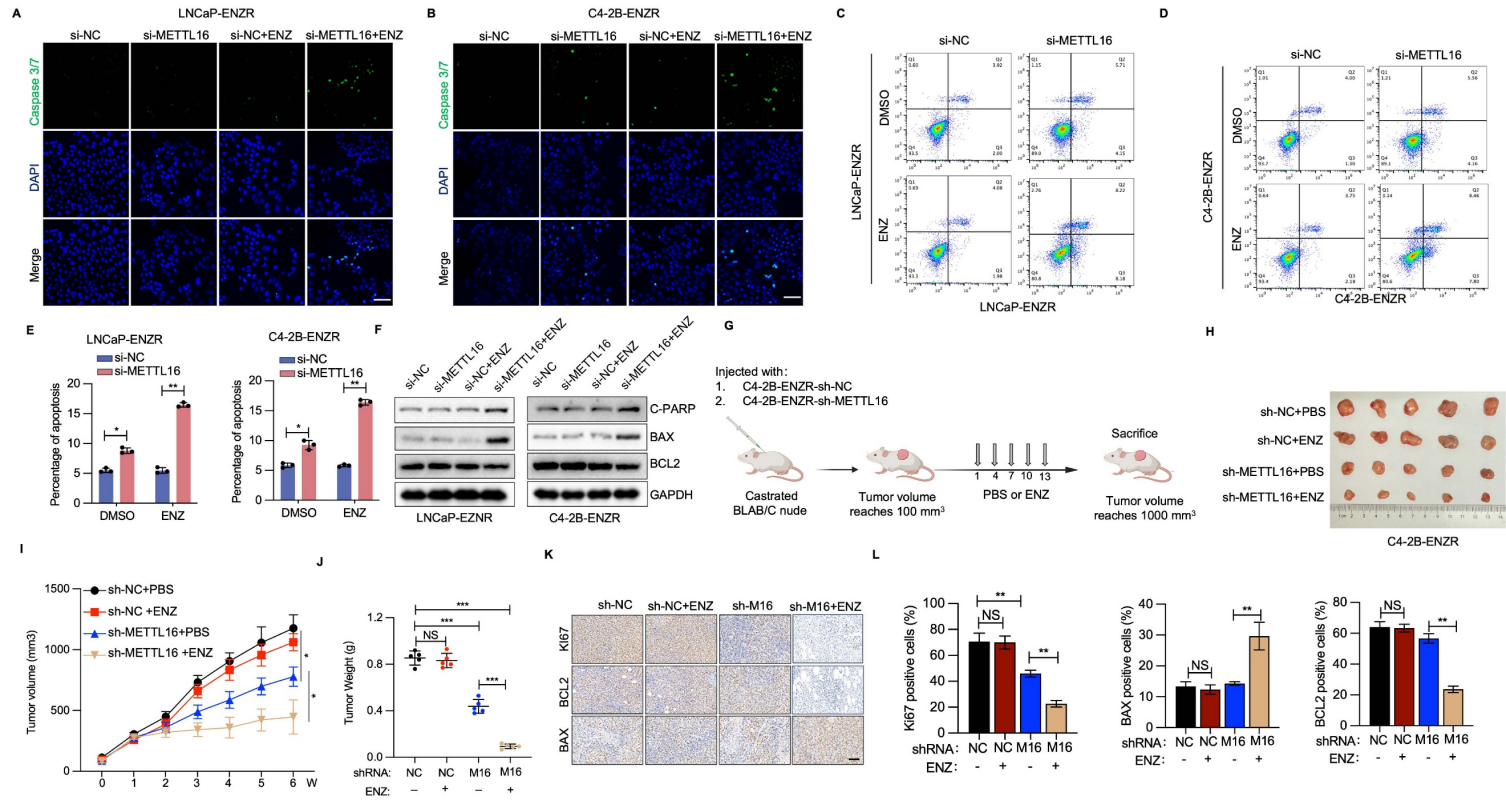
** METTL16 deficiency and ENZ treatment synergistically trigger apoptotic mechanisms.** (A-B) Fluorescence signal intensity of caspase 3/7 and DAPI detected via immunofluorescence staining. (C-E) Apoptosis cellular assessment through flow cytometry for cell populations transfected with METTL16 siRNAs or control siRNA under DMSO or ENZ exposure conditions. (F) Protein expression patterns of cleaved PARP, BAX, and BCL-2 in siNC or siMETTL16 cellular environments post-ENZ treatment. (G) Methodological approach for xenograft mouse model establishment utilizing C4-2B-ENZR cells. (H-J) Effects of METTL16 knockdown combined with ENZ treatment on tumor growth in a subcutaneous xenograft model. Tumor dimensions were monitored at predetermined intervals, with tumor specimens harvested and quantified following animal sacrifice. (K-L) Immunohistochemical visualization and quantitative analysis of Ki67, BCL2 and BAX. Scale bar, 50 µm. *P < 0.05, **P < 0.01, ***P < 0.001.

Apoptosis induction represents a primary mechanism through which ENZ suppresses PCa^31^, ^32^. Therefore, resistance to ENZ-induced apoptosis serves a critical function in ENZR development. To examine whether METTL16 inhibition enhances apoptosis in ENZR PCa cells after ENZ intervention, immunofluorescent staining for caspase 3/7 was conducted in si-NC and si-METTL16 PCa cells with or without ENZ treatment. A notable elevation in caspase 3/7 staining was detected in the combination treatment group of si-METTL16 plus ENZ, relative to single-agent treatment (Figure 5A, 5B). Supporting these findings, Annexin V-FITC and PI staining revealed that the combination intervention of si-METTL16 and ENZ markedly induced apoptosis in ENZR PCa cells (Figure 5C-E). WB verified the considerable upregulation of the apoptotic marker cleaved PARP following combination treatment with si-METTL16 and ENZ in C4-2R-ENZR and LNCaP-ENZR cells (Figure 5F).

ENZ suppresses PCa through regulation of BCL2 signaling^32^, ^33^. Analysis of BCL2 family proteins demonstrated that BCL-2 levels (an anti-apoptotic protein) were diminished in cells subjected to si-METTL16 and ENZ treatment, whereas BAX (a pro-apoptotic protein) and C-PARP exhibited significant upregulation (Figure 5F). These observations suggest that inhibiting METTL16 potentiates ENZ-triggered programmed cell death by interfering with protective BCL2 signaling mechanisms.

To evaluate the potential therapeutic impact of METTL16 targeting in ENZR PCa xenografts, C4-2B-ENZR cells stably knockdown of METTL16 or control were subcutaneously injected into nude mice (Figure 5G and Figure S2E). Consistent with initial hypotheses, the integrated shMETTL16 and ENZ intervention markedly diminished xenograft tumor progression and mass, exhibiting the most pronounced inhibition of tumorigenic potential versus individual shNC, ENZ, or shMETTL16 treatment protocols (Figure 5H-J). IHC examination of tumor specimens showed no notable alterations in BCL2 and Ki67 protein expression in the shNC or ENZ treatment groups, whereas substantial decreases were detected in the combined shMETTL16 and ENZ treatment group (Figure 5K, 5J). Conversely, BAX expression was markedly elevated in the combination treatment group (Figure 5K, 5J). These results further support the potential of METTL16 as a promising target to overcome ENZR in PCa.

### EEF1A1 is the m6A-dependent target of METTL16 in ENZR cells

**Figure 6:**
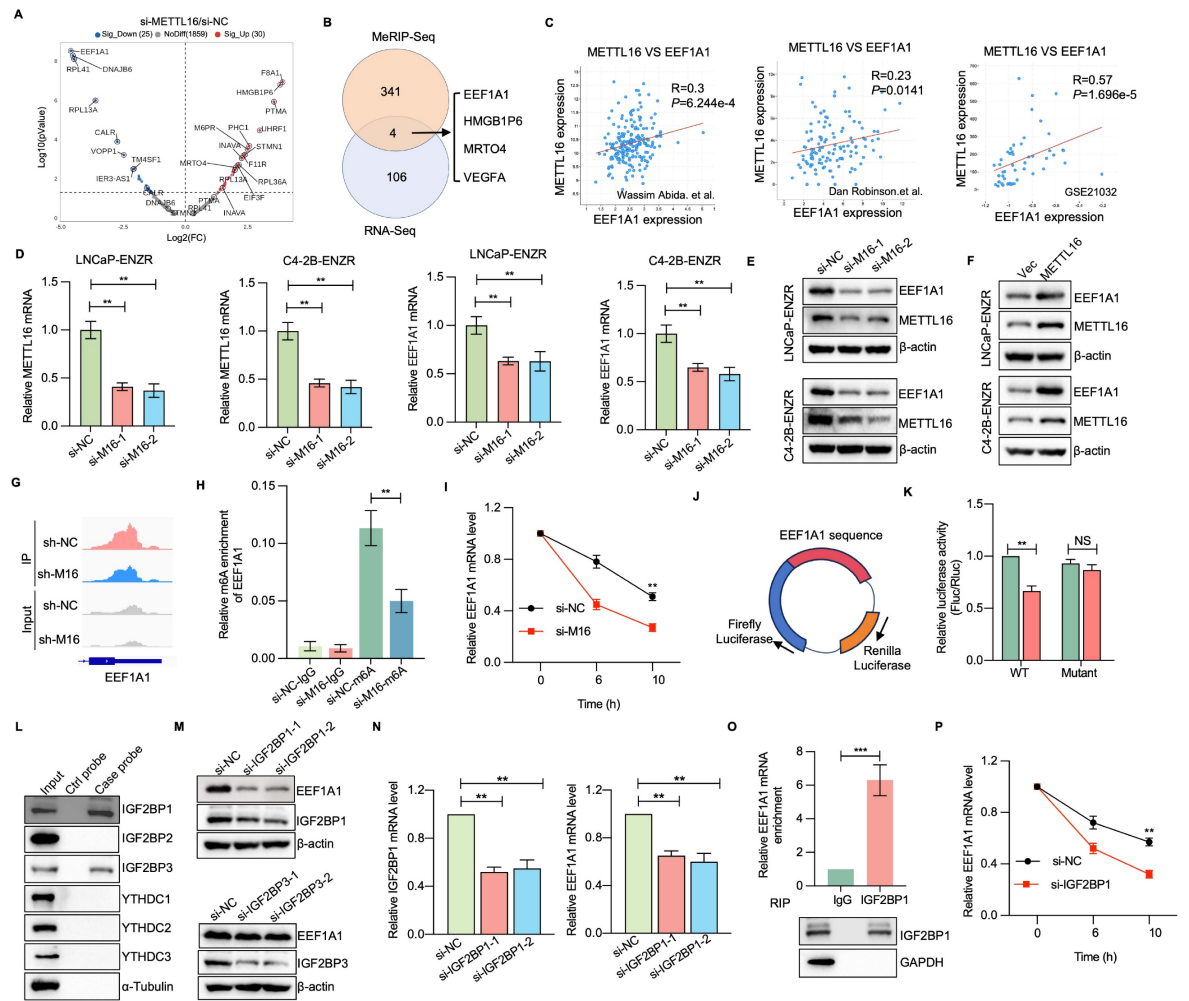
** Identification of EEF1A1 as a direct METTL16 target.** (A) Differential methylation gene expression profile visualized through volcano plot after METTL16 knockdown. (B) Four potential METTL16 target genes identified via RNA-sequencing and MeRIP sequencing intersection. (C) Expression correlation between METTL16 and EEF1A1 in publicly available PCa databases. (D) RT-qPCR analysis of EEF1A1 mRNA expression in the indicated PCa cells. (E-F) EEF1A1 protein levels in PCa cells subjected to METTL16 knockdown and overexpression were assessed through WB analysis. (G) m6A peak visualization of m6A-seq data for EEF1A1 transcripts in PCa cells following METTL16 knockdown compared to control conditions. (H) Relative m6A levels of EEF1A1 mRNA in PCa cells following METTL16 knockdown were evaluated and normalized to input via MeRIP-qPCR. (I) EEF1A1 mRNA stability evaluation in METTL16 knockdown cells post-actinomycin D treatment. (J-K) Luciferase reporter assay using pmirGLO vector with wild-type and mutant sequences. Luciferase activity measured in HEK293T cells under METTL16 knockdown, with Renilla luciferase as internal control. (L) Immunoblotting of RNA pull-down assays performed with the indicated m⁶A reader proteins. (M-O) Assessment of EEF1A1 expression following IGF2BP1/3 knockdown in C4-2B cells. (P) RIP-qPCR analysis of EEF1A1 mRNA enrichment with IGF2BP1 immunoprecipitation. (Q) Evaluation of EEF1A1 mRNA stability in cells with IGF2BP1 knockdown. *P < 0.05, **P < 0.01, ***P < 0.001.

To investigate the molecular mechanisms linked to METTL16-mediated ENZR, we conducted MeRIP-sequencing and RNA-sequencing in C4-2B-ENZR cells featuring METTL16 knockdown and their respective control. Subsequent MeRIP-sequencing analysis revealed 8,570 and 7,603 m6A peaks in the control and METTL16-deficient cellular populations, respectively (Figure S3A). The distribution of m6A peaks demonstrated enrichment in the coding sequence and 3' UTR, with a pronounced density peak surrounding the stop codon (Figure S3B). The m6A consensus motif, featuring CAG-containing and/or AG-rich sequences, was predominant in the detected peaks, aligning with previous findings^23^, ^34^. Additionally, RNA-sequencing data revealed that METTL16 depletion induced upregulation of 30 genes and downregulation of 25 genes, as depicted in Figure 6A. Four differentially expressed genes and peaks exhibited overlapping characteristics (Figure 6B). Notably, EEF1A1 displayed the most markedly diminished mRNA level in si-METTL16 cells versus the control cells. Subsequent cross-database analysis across three public PCa datasets confirmed a marked positive correlation between METTL16 and EEF1A1 expression^35^-^37^ (Figure 6C). GEO dataset examination indicated EEF1A1 upregulation in PCa tumors and CRPC versus normal adjacent tissues or CSPC (Figure S3). Given that EEF1A1 is a known negative regulator of apoptosis^38^, ^39^, it was hypothesized that METTL16 suppresses ENZ-induced apoptosis in an EEF1A1-dependent manner. Verification of this hypothesis showed that METTL16 knockdown resulted in reduced EEF1A1 expression at both the mRNA and protein levels (Figure 6D, 6E). Furthermore, MeRIP-seq data identified an m6A peak in the EEF1A1 mRNA, which was reduced following METTL16 knockdown (Figure 6G). MeRIP-qPCR analysis validated that m6A-modified EEF1A1 mRNA was markedly diminished after METTL16 knockdown (Figure 6H). As anticipated, RNA stability assays revealed that METTL16 suppression substantially decreased EEF1A1 mRNA stability, consequently reducing its overall abundance (Figure 6I).

Fancm, Brca2, U6 snRNA, and MAT2A mRNA contain METTL16-target consensus sequences (5'-ACAGAR-3') located within structured RNA regions^34^. This study explored whether EEF1A1 transcripts also harbor similar structured motifs. Such a motif was identified within the coding sequences of EEF1A1 (Figure S3C). To evaluate the impact of METTL16-mediated m6A modification on EEF1A1 expression, a luciferase reporter construct was designed, incorporating either WT or mutated m6A sites within the EEF1A1 coding sequence (Figure 6J). Luminescence measurements revealed that silencing METTL16 markedly diminished reporter gene activity when utilizing the wild-type EEF1A1 sequence, whereas no substantial alteration was observed with the mutated variant (Figure 6K). The experimental outcomes suggest a direct regulatory link between METTL16 and EEF1A1.

IGF2BP proteins, along with YTHDC1/2/3, are recognized for their ability to identify m6A modifications and enhance the stability of the associated mRNAs. To identify the specific m6A reader responsible for EEF1A1 methylation, an RNA pull-down assay was performed to isolate EEF1A1-interacting readers. Results indicated that EEF1A1 mRNA displayed preferential interaction with IGF2BP1 and IGF2BP3 (Figure 6L). Interestingly, the depletion of IGF2BP1, but not IGF2BP3, significantly reduced both EEF1A1 mRNA and protein expression in PCa cells (Figure 6M, 6N). Furthermore, RNA immunoprecipitation (RIP) experiments substantiated that IGF2BP1 directly interacts with EEF1A1 mRNA (Figure 6O). Consistent with expectations, RNA stability investigations demonstrated that the depletion of IGF2BP1 markedly diminished the stability of EEF1A1 mRNA (Figure 6P). Collectively, these observations propose that METTL16 introduces m6A modifications to EEF1A1 transcripts, which are subsequently recognized by IGF2BP1, thereby enhancing mRNA stability in PCa cells.

### The METTL16/m6A/ EEF1A1 axis suppresses ENZ-induced apoptosis of ENZR PCa cells

**Figure 7:**
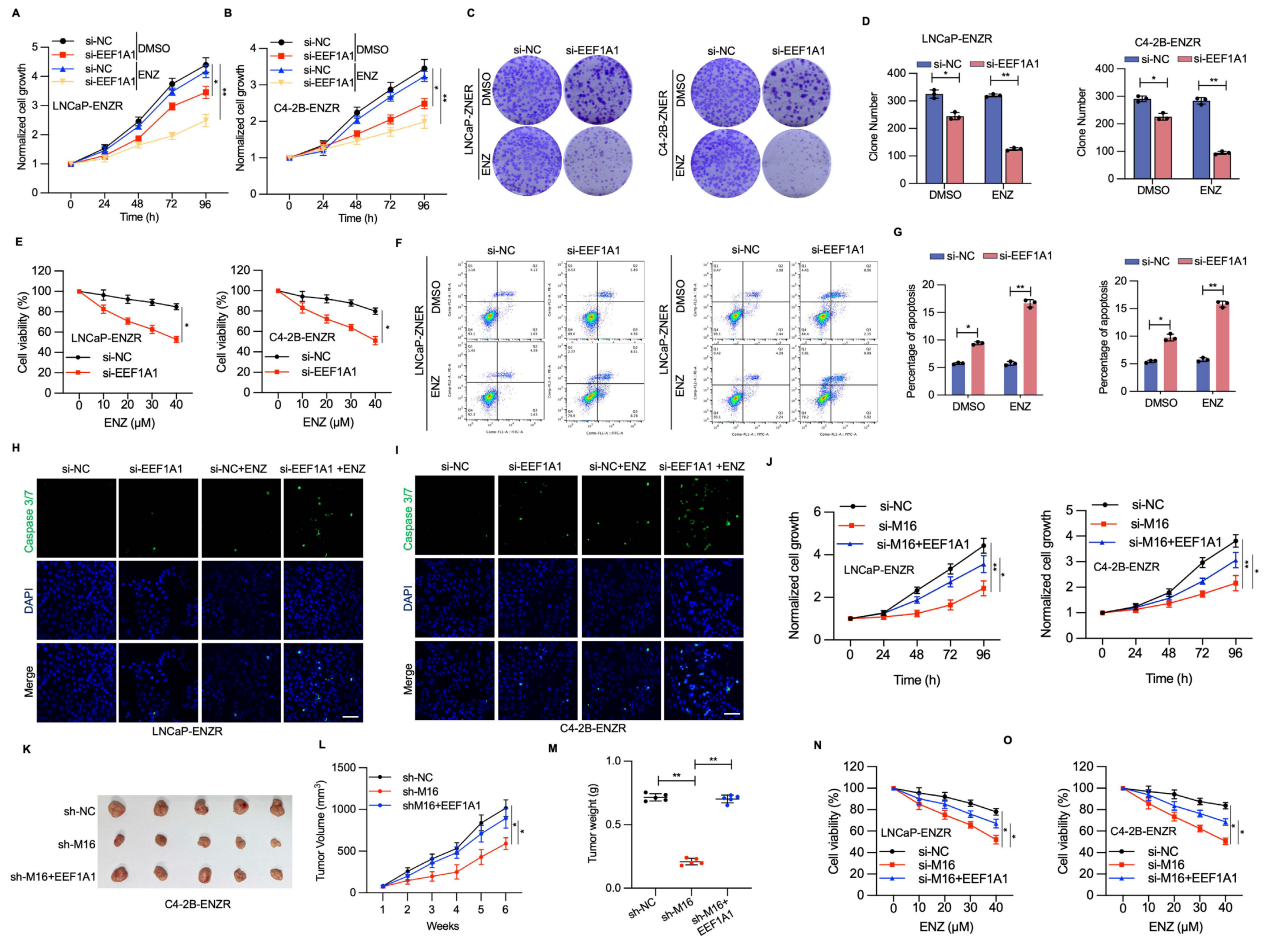
** The METTL16-EEF1A1 axis confers ENZR.** (A-D) Cell proliferation assessment was conducted using CCK-8 assay and colony formation assay in LNCaP-ENZR and C4-2B-ENZR cells after transfection with NC or EEF1A1 siRNA. (E) Cell viability was assessed in designated cell lines during ENZ treatment. LNCaP-ENZR and C4-2B-ENZR cells underwent transfection as specified and were subjected to escalating ENZ concentrations for 3 days. Cell viability was ascertained through CCK-8 assays. (F-G) Apoptosis detection via flow cytometry in cells transfected with EEF1A1 siRNAs or negative control following DMSO or ENZ treatment. (H-I) Fluorescence intensity of caspase 3/7 and DAPI was examined through immunofluorescence staining assays. (J) CCK-8 assay was conducted to assess the proliferation of LNCaP-ENR or C4-2B-ENR siM16 cells following EEF1A1 transfection. (K-M) Xenografts obtained from C4-2B-ENZR-shM16 or C4-2B-ENZR-shM16-OE-EEF1A1 cells and respective controls. Tumor dimensions were recorded at specified intervals, and tumors were extracted and measured following mouse euthanasia. (N-O) Cell viability was determined in designated cell lines during ENZ treatment. LNCaP-ENZR and C4-2B-ENZR cells underwent transfection as specified and exposure to graduated ENZ doses for 3 days. Cell viability was assessed through CCK-8 assays. Scale bar, 50 µm. *P < 0.05, **P < 0.01, ***P < 0.001.

To assess the potential function of EEF1A1 in ENZR development, RNA interference techniques were utilized to diminish EEF1A1 expression within LNCaP-ENZR and C4-2B-ENZR cells. EEF1A1 reduction led to a marked reduction in cell proliferation and colony formation across both cell lines (Figure 7A-D). Cell viability experiments showed that EEF1A1 knockdown considerably restored ENZR cell sensitivity to ENZ (Figure 7E). Additionally, Annexin V-FITC and PI staining indicated that the combined intervention of si-EEF1A1 and ENZ triggered marked apoptosis in ENZR PCa cells (Figure 7F, 7G). A notable elevation in caspase 3/7 staining was also detected with the combination treatment, relative to individual-agent treatment (Figure 7H, 7I). Subsequently, the function of EEF1A1 in METTL16-mediated PCa progression was examined by upregulating EEF1A1 in PCa cells with METTL16 knockdown. EEF1A1 upregulation substantially reversed the suppressive impact of METTL16 knockdown on cell proliferation both *in vitro* and *in vivo* (Figure 7J-M). As expected, ENZR was reduced in si-METTL16 cells and was partially restored upon re-expression of EEF1A1 (Figure 7N, 7O). These results suggest that METTL16's role in promoting ENZR is partially dependent on EEF1A1.

### UFL1-METTL16-EEF1A1 axis triggers PCa progression

**Figure 8:**
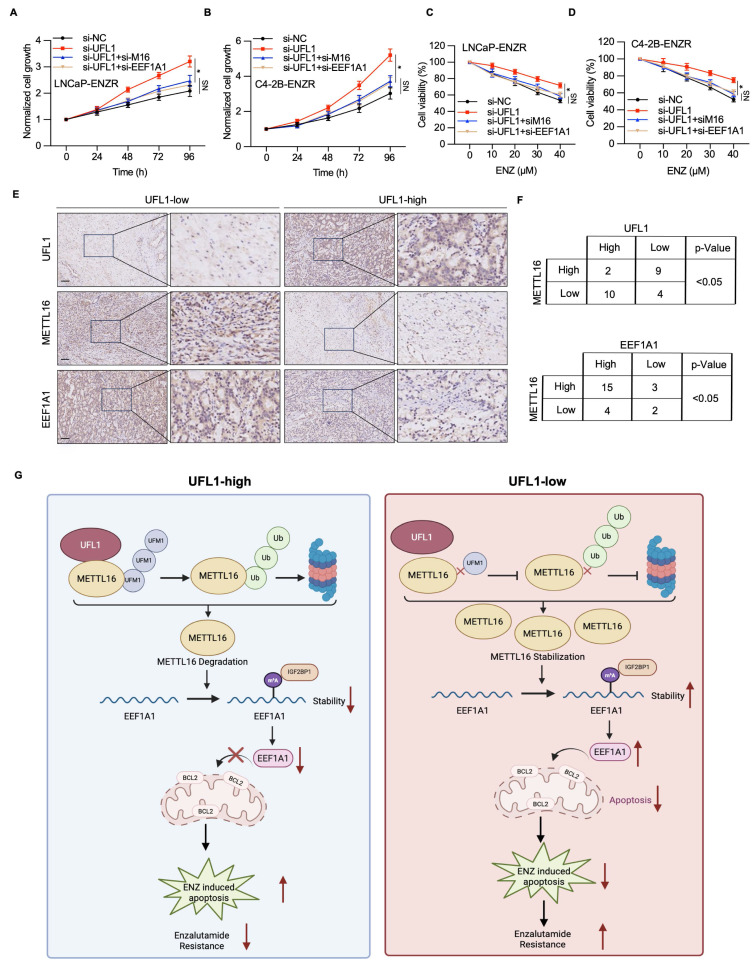
** UFL1/METTL16/EEF1A1 is involved in prostate cancer progression.** (A-B) CCK-8 assay was conducted to measure the proliferation of LNCaP-ENR or C4-2B-ENR siUFL1 cells transfected with EEF1A1 or METTL16 siRNA. (C-D) Cell viability was measured in the indicated cell lines under enzalutamide treatment. LNCaP-ENZR and C4-2B-ENZR cells were transfected as indicated and treated with titrated doses of enzalutamide for 3 days. Cell viability was quantified by the CCK-8 assays. (E-F) Protein expression of UFL1, METTL16, and EEF1A1 was examined in 25 patients with prostate cancer using IHC. The staining intensity was scored as low (- or +) and high (++) in three different areas of each tumor section. χ^2^ test = Chi-square test. (G) Proposed model illustrating the mechanisms of the UFL1/METTL16/EEF1A1 axis in prostate enzalutamide resistance. Scale bar, 50 µm. *P < 0.05, **P < 0.01, ***P < 0.001.

To elucidate the mechanistic involvement of METTL16/EEF1A1 in UFL1-mediated ENZR in PCa, METTL16 or EEF1A1 expression was downregulated using siRNA in si-UFL1 PCa cells, and their sensitivity to ENZ was assessed. The results showed that UFL1 loss-induced ENZR was attenuated by METTL16 or EEF1A1 knockdown (Figure 8A-D). Finally, to investigate the potential link between UFL1, METTL16, EEF1A1, and PCa progression, the protein expression of these factors was analyzed in 25 PCa tissue samples using IHC. IHC analysis demonstrated that elevated UFL1 expression correlated with relatively diminished METTL16 and EEF1A1 expression (Figure 8E, 8F), indicating that METTL16 and EEF1A1 protein levels exhibit a negative correlation with UFL1 in PCa patients. Collectively, these findings indicate that the UFL1-METTL16-EEF1A1 axis contributes markedly to PCa progression.

## Discussion

The next-generation AR pathway inhibitor ENZ has demonstrated efficacy in extending survival among PCa patients. Nevertheless, the emergence of ENZR continues to be unavoidable, and the fundamental mechanisms underlying this resistance remain incompletely elucidated^3^, ^4^. In this study, the loss of UFL1 was identified as a key contributor to ENZR in PCa cells. Specifically, UFL1 deficiency stabilizes METTL16 protein, resulting in increased expression of EEF1A1 and reduced ENZ-induced apoptosis in resistant cells. Mechanistically, UFL1 promotes the UFMylation of METTL16, which facilitates its ubiquitination and subsequent proteasomal degradation. Stabilized METTL16, in turn, enhances the m6A modification of EEF1A1 mRNA—a known anti-apoptotic factor—thus elevating its stability and expression through an IGF2BP1-dependent mechanism. Collectively, these findings uncover a previously unrecognized UFL1-METTL16-EEF1A1 signaling axis that mediates ENZR and highlight UFL1 as a potential strategic intervention to address ENZR in CRPC.

METTL16 has been implicated in promoting tumor initiation and progression in various cancers, as well as conferring chemoresistance in hepatocellular carcinoma and colorectal cancer^27^, ^40^-^44^. Despite existing knowledge, the specific implications for PCa, especially within ENZR contexts, have not been comprehensively investigated. This investigation reveals that METTL16 serves a pivotal function in facilitating prostate adenocarcinoma cellular proliferation and progression, ultimately contributing to the emergence of ENZR. A significant upregulation of METTL16 was observed in ENZR cell lines and clinical PCa tissues, with high METTL16 expression strongly correlating with poor prognostic outcomes. Functionally, METTL16 targeting markedly enhanced the responsiveness of ENZR cells to ENZ treatment, both *in vitro* and *in vivo*, indicating that METTL16 serves a vital function in maintaining resistance phenotypes. Mechanistically, integrated MeRIP-seq and RNA-seq analyses revealed *EEF1A1*—a known anti-apoptotic gene^38^—as a primary downstream target of METTL16. The data demonstrate that METTL16 directly associates with* EEF1A1* mRNA, promoting its m6A modification and elevating its mRNA stability, which ultimately results in elevated EEF1A1 protein levels. Notably, IGF2BP1, but not other m6A reader proteins, was identified as the key mediator that recognizes the m6A-modified *EEF1A1* transcript and facilitates its post-transcriptional regulation. This METTL16-IGF2BP1-EEF1A1 axis appears to be a critical driver of ENZR through the suppression of apoptosis. Specifically, elevated EEF1A1 levels were associated with increased BCL-2 activation, inhibiting ENZ-induced apoptotic signaling. Thus, by post-transcriptionally regulating EEF1A1 expression in an m6A-dependent manner, METTL16 serves a pivotal function in controlling ENZR progression.

UFM1 (ubiquitin-like modifier 1) represents a newly discovered ubiquitin-like modification that serves a crucial function in diverse biological processes and disease advancement^9^, ^10^. Aberrant regulation of UFMylation has been connected to the progression of diverse cancers^45^-^47^. The ufmylation of estrogen receptor-α (ERα) and activating signal cointegrator 1 (ASC1) facilitated the growth of breast cancer^48^-^50^. In pancreatic adenocarcinoma, the UFMylation of RPL10 enhances cell proliferation and stemness, chiefly via upregulating the transcription factor KLF4^51^. Furthermore, through ArpC4 UFMylation, UFL1 operates as a positive regulator of metastasis in lung cancer^52^. Despite the oncogenic activity of UFL1 in breast cancer, pancreatic adenocarcinoma, and lung cancer, it may act as a tumor suppressor role in hepatocellular carcinoma and Ovarian, by modulating NF-kB signaling^53^, ^54^. In this study, we found UFL1 interacts with and catalyzes the UFMylation of METTL16, destabilizes METTL16 by synergizing with its ubiquitination. Loss of UFL1 promoted ENZR by enhancing EEF1A1 expression in an METTL16-dependent manner, thereby inhibiting ENZ-induced apoptosis. Considering that the biological pathways regulated by the UFL1-METTL16 axis are therapeutically relevant, it would be of interest to develop small molecules or peptides that disrupt their interaction.

In conclusion, this investigation revealed the UFL1-METTL16-EEF1A1 axis as an essential regulatory mechanism of ENZR in PCa cells, underscoring its promising potential as a strategic therapeutic approach for overcoming ENZR in PCa.

## Materials and Methods

### Cell culture and treatments

Human PCa cell lines (LNCaP and C4-2B) along with HEK293T cells were procured from the American Type Culture Collection (ATCC) and cultured per the supplier's protocols. The enzalutamide-resistant cells (LNCaP-ENR and C4-2B-ENR) are generated in-house. Specifically, two drug-resistant cell lines (LNCaP-ENR and C4-2B-ENR) were established by gradually increasing enzalutamide concentrations from 5 to 40 µM over three months, followed by continuous culture in medium containing 40 µM enzalutamide for an additional four months. The established resistant cells are maintained in culture medium with 20 μM enzalutamide. All cell cultures were maintained in a humidified atmosphere comprisinging 5% CO_2_ at 37°C. ENZ was procured from Med Chem Express and prepared as per the supplier's protocols.

### Plasmids, siRNA, and transfection

Full-length cDNA sequences of UFL1 or METTL16 underwent cloning into pcDNA3.1, pET30a, or CMV-puro lentiviral plasmids. Plasmids expressing His-UFM1, MYC-UFSP2, HA-METTL16, FLAG-UFL1, and METTL16 mutants were cloned into the pCDNA3.1 vector. METTL16 knockdown lentivirus plasmids (shMETTL16-1, shMETTL16-2) and their corresponding control were purchased from GENRTALl BIOL (Chuzhou, China). All constructs utilized in this investigation underwent verification through DNA sequencing. The virus particles were produced using HEK-293T cells by co-transfection with pMD2G and pSPAX2 (Addgene, USA). For each 6-cm dish of cells at 60% confluence, 1 mL virus was added along with 8 μg/mL polybrene (Sigma). After puromycin selection, knockdown or overexpression efficiency was confirmed by Western blot analysis. Plasmids and siRNA were introduced into the designated cells utilizing Lipofectamine 2000 per the supplier's protocols (Invitrogen, Carlsbad, CA).

### Colony formation assay and CCK-8 assay

For the colony formation assay, cells (~1000) were plated in triplicate (6-well plates) per condition and permitted to attach for at least 16 hours. Following 10-14 days, plates underwent fixation, staining with Crystal Violet, and enumeration. In the cell proliferation assay, 5 × 10^3^ cells/well were plated into 96-well plates. Following cell attachment, 10 μL of CCK-8 was introduced to each well at 24, 48, 72, and 96 hours, and absorbance was determined at 450 nm.

### Immunofluorescence

Cells cultured on coverslips underwent fixation with 4% paraformaldehyde (PFA) in PBS for 15 minutes, subsequently permeabilized using 0.5% Triton X-100 in PBS for 10 minutes. The fixed cells received incubation with primary antibodies for 2 hours and secondary antibodies for 1 hour, both treatments conducted in 1% BSA in PBS comprising 0.25% Triton X-100 at ambient condition. DNA was counterstained with DAPI for 3-5 minutes. Data analysis was performed using ImageJ.

### WB and immunoprecipitation

Cells underwent lysis employing RIPA buffer (Beyotime, Shanghai, China) in combination with protein loading buffer (Solarbio, Beijing, China). The extracted proteins underwent separation via SDS-polyacrylamide gel electrophoresis and were subsequently transferred to PVDF membranes. The membranes received treatment with designated primary antibodies, after which they were exposed to HRP-conjugated secondary antibodies. For immunoprecipitation procedures, cell lysis occurred in IP buffer (Beyotime, Shanghai, China), and the resulting lysates received antibody incubation for 12 hours at 4 °C, after which Protein G Sepharose was introduced for an extra hour. Beads underwent washing with lysis buffer, followed by boiling in SDS sample buffer, and subsequent analysis through immunoblotting. The main antibodies used in WB were as follows: METTL16 (Sigma, HPA020352, 1:2000), METTL16 (abconal, A15894, 1:4000), GAPDH (abconal, AC001, 1:5000), Flag (CST, 14793, 1:5000 ), Flag (Sigma, F3165, 1:5000), HA (abconal, AE008, 1:5000), Myc (Abcam, ab32072, 1:5000), UFL1 (F5P6L, Cell Signaling Technology, 1:1000; HPA030559, Sigma,1:1000), UFSP2 (16999-1-AP, Proteintech, 1:1000), and EEF1A1 (2551, Cell Signaling Technology,1:1000).

### Apoptosis assays

To examine cellular apoptosis levels, cells were plated in 6-well plates at 2 × 10^5^ cells per well. Following 48 hours of adherence, cells received treatment with DMSO or ENZ (20 μM) for 24 hours. Apoptosis detection was executed employing Annexin V-FITC Apoptosis Detection Kits (KGA1102-20) per the supplier's protocols. Flow cytometry analysis and FlowJo software were employed to quantify and evaluate apoptosis rates. Furthermore, Caspase-3/7 Green ReadyProbes™ (C11061-2, Invitrogen) were utilized for apoptosis detection in cells, with fluorescent signals visualized employing a fluorescence microscope.

### RNA extraction and real-time RT-PCR

RNA extraction was executed utilizing Trizol (Invitrogen), and quantitative real-time PCR (qRT-PCR) was executed as previously established protocols, employing GAPDH as a normalization control. For RNA stability assays, cells were plated in 6-well plates overnight. RNA decay rates were assessed by treating cells with actinomycin D (HY-17559, MedChemExpress) at 5 μg/mL concentration and collecting the cells at designated time points. PCR primers were as follows: GAPDH, forward 5′-TGCACCACCAACTGCTTAGC-3′, reverse 5′-GGCATGGACTGTGGTCATGAG-3′; EEF1A1, forward 5′-AAGGATGTTCGTCGTGGCAA, reverse 5′-GCCGTGTGGCAATCCAATAC; METTL16, forward 5′-AGGGAGTAAACTCACGAAATCCT, reverse 5′-AACCCCTTGTATGCGAAGCTC; IGF2BP1, forward 5′-TAGTACCAAGAGACCAGACCC, reverse 5′-GATTTCTGCCCGTTGTTGTC.

### MeRIP-sequencing

Total RNA was procured from SW620 cell populations following transfection with shMETTL16-2 or shNC, employing the TRIzol reagent methodology. Poly (A) + RNA underwent precise purification and subsequent fragmentation utilizing the NEB Next poly (A) mRNA Magnetic Isolation Kit (New England Biolabs, UK). Subsequent MeRIP-sequencing and comprehensive data analysis were conducted by Genesky Biotechnologies Inc. (Shanghai, China). Suitably prepared samples were subjected to library pooling and sequencing procedures utilizing Illumina HiSeq 2500 instrumentation. Following stringent quality filtering steps, primary sequence data were aligned to the human genome (GRCh37/hg19) using HISAT2 software (v2.0.5), with the resulting outputs then analyzed through bioinformatics and statistical methodologies.

### RNA immunoprecipitation

Cell lysates underwent incubation with 1 μg of specific antibodies targeting IgG or IGF2BP1 at 4 °C overnight for precipitating m6A-conjugated RNAs within the reaction buffer. Subsequently, 20 μL of washed magnetic beads was introduced to each reaction and maintained at 4 °C for 2 hours. Post-incubation, the samples underwent three consecutive phosphate-buffered saline (PBS) washing cycles. Subsequently, target RNAs present in the immunoprecipitation complex underwent extraction and purification procedures for subsequent quantitative analysis via qRT-PCR. The obtained RNA enrichment levels were subsequently standardized versus the input sample.

### PCa tissue specimens and immunohistochemistry

PCa specimens were procured from patients at The Third Affiliated Hospital of Soochow University during the period from January 2016 to December 2018. Informed consent was secured from each patient, excluding those individuals who had undergone systemic or local therapy. The research protocol received approval from the Third Affiliated Hospital of Soochow University. IHC was conducted to evaluate target protein expression according to established protocols. IHC images were acquired employing an Olympus BX63 microscope, and immunostaining was evaluated by pathologists under blinded conditions.

### Xenograft animal model

Male BALB/c nude mice (4-6 weeks old) were procured from the Third Affiliated Hospital of Soochow University. For *in vivo* experiments, 6-week-old male nude mice underwent castration procedures. C4-2B-ENZR cells stably transfected with shMETTL16 or UFL1-overexpressing lentivirus, along with their corresponding negative controls, were prepared by digestion and subsequent washing. Subsequently, the mice received anesthetic treatment, and cells were subcutaneously implanted with Matrigel premixed at a 1:2 ratio into the right rear flank. Upon achieving a tumor volume of 100 mm³, mice were orally administered with 10 mg/kg ENZ or PBS. Tumor measurements were systematically tracked on a weekly basis following injection, calculated utilizing the established mathematical equation 0.5 × a² × b, wherein "a" and "b" signify the respective short and extended tumor diameters. At the research's terminal phase, all experimental subjects underwent ethical euthanasia, after which tumor specimens were extracted, precisely quantified, and subsequently prepared for comprehensive histological examination. This animal study received approval from the Third Affiliated Hospital of Soochow University.

### Publicly available data

Datasets from GSE44927, GSE116918, GSE35988, and GSE8511 were procured from the GEO database (http://www.ncbi.nlm.nih.gov/geo), and UFL1 and METTL16 expression levels in these datasets were examined across the specified groups. RNA-seq data from The Cancer Genome Atlas (TCGA) were procured from (http://gdac.broadinstitute.org/).

### Statistical analysis

The data were analyzed using GraphPad Prism software and presented as the mean ± SD(SD) with at least three independent experiments. Comparisons between two groups were examined employing the two-tailed Student's t-test for parametric data or the Mann-Whitney test for non-parametric data. Statistical significance was considered at a P-value of less than 0.05 in all cases.

## Supplementary Material

Supplementary figures.
