# Supplementary material for: Loss of UFL1 confers enzalutamide resistance of prostate tumors by regulating METTL16-mediated m6A modification of EEF1A1 mRNA
Source: Int J Biol Sci. 2026 Jan 1;22(3):1306–21. doi: 10.7150/ijbs.124214 (PMC12837662; doi:10.7150/ijbs.124214)
Supplement: Supplementary file 1 — Supplementary figures. [file ijbsv22p1306s1.pdf]

Figure S1

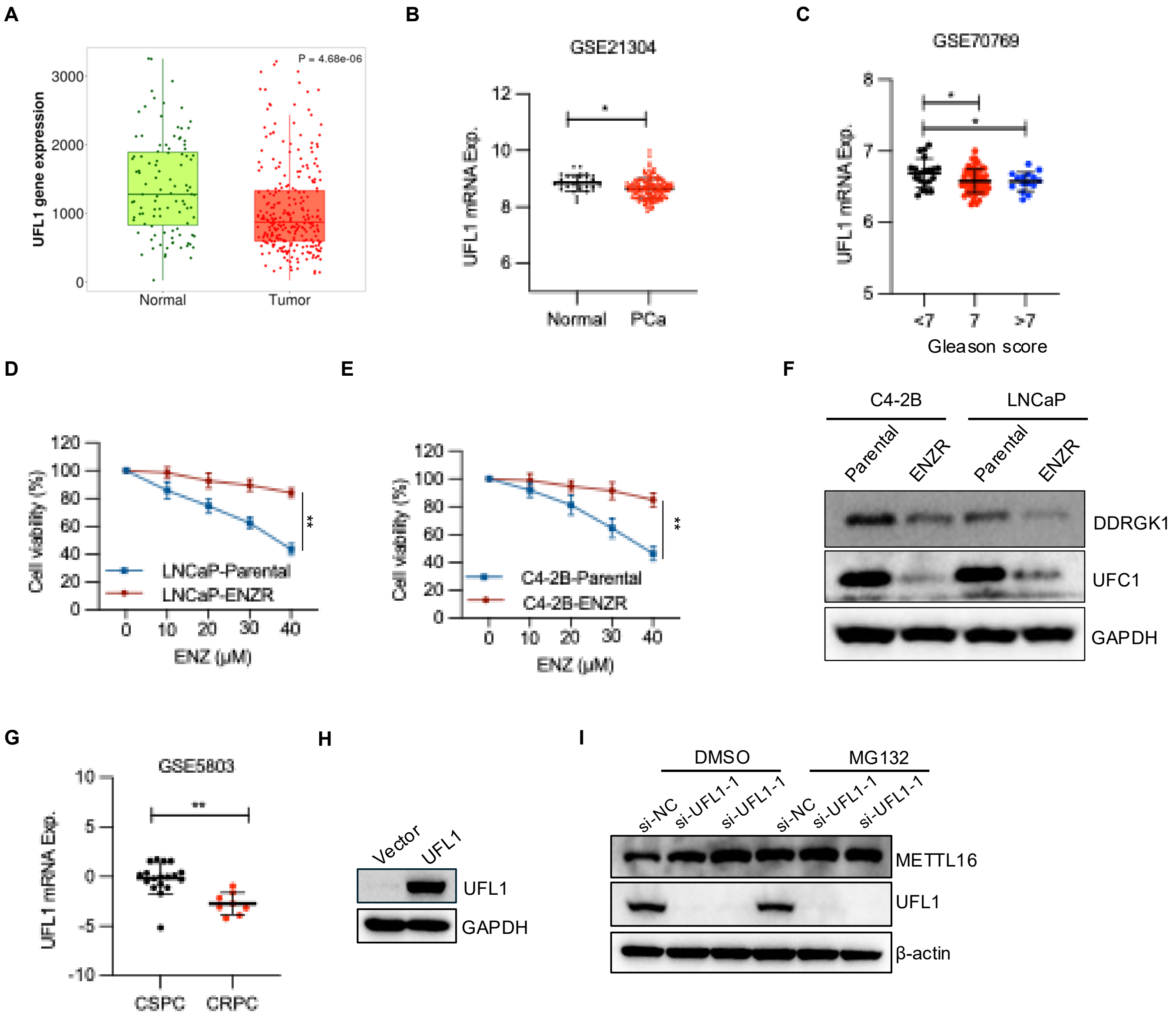

(A) Relative mRNA expression of UFL1 in prostate cancer samples from TNMplot database; (B) Relative mRNA expression of UFL1 in normal and prostate cancer samples from GEO database; (C) Analysis of UFL1 expression in GSE70769 datasets. UFL1 expression distributed in PCa with different Gleason score; (D-E) Cell viability was evaluated in designated cell lines during ENZ treatment. (F) DDRGK1 and UFC1 levels in PCa cell lines. LNCaP-Parental, LNCaP-ENZR, C4-2B-Parental, and C4-2B-ENZR cells were procured and examined by Western blotting. (G) Relative mRNA expression of UFL1 in CSPC and CRPC samples from GEO database. (H) C4-2B-ENZR cells were stably transfected UFL1-overexpressing lentivirus or a control plasmid. Western blotting was performed to detect the expression levels of UFL1. (I) Indicated cells were treated with MG132 (20 μM) followed by western blot with anti-METTL16.*P< 0.05, **P<0.01.

Figure S2

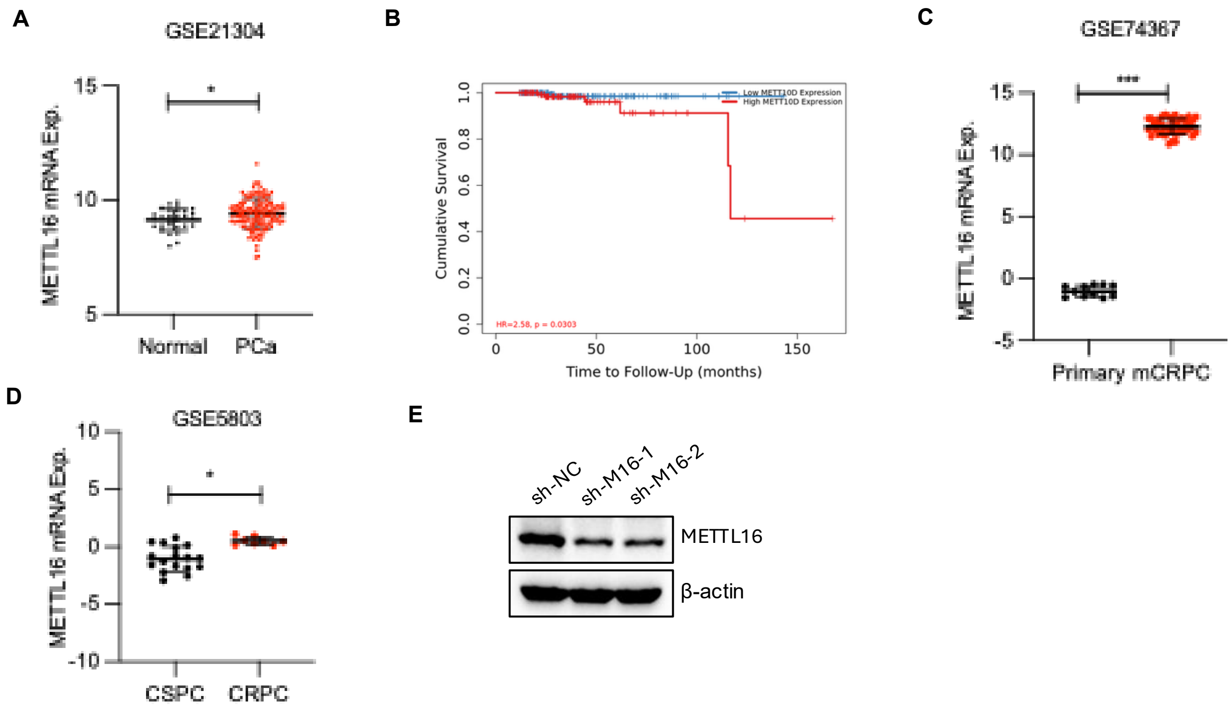

(A) Relative mRNA expression of METTL16 in prostate cancer samples from GEO database; (B) Kaplan–Meier survival analysis of PCa cases according to high and low METTL16 expression levels; (C) normal and prostate cancer samples from GEO database; (C) Analysis of METTL16 expression in GSE74367 datasets; (D) Relative mRNA expression of METTL16 in CSPC and CRPC samples from GEO database. (E) Western blot analysis of METTL16 protein in C4-2B-ENZR cells with METTL16 knockdown by specific shRNAs.*P< 0.05, **P<0.01, ***P<0.001.

Figure S3

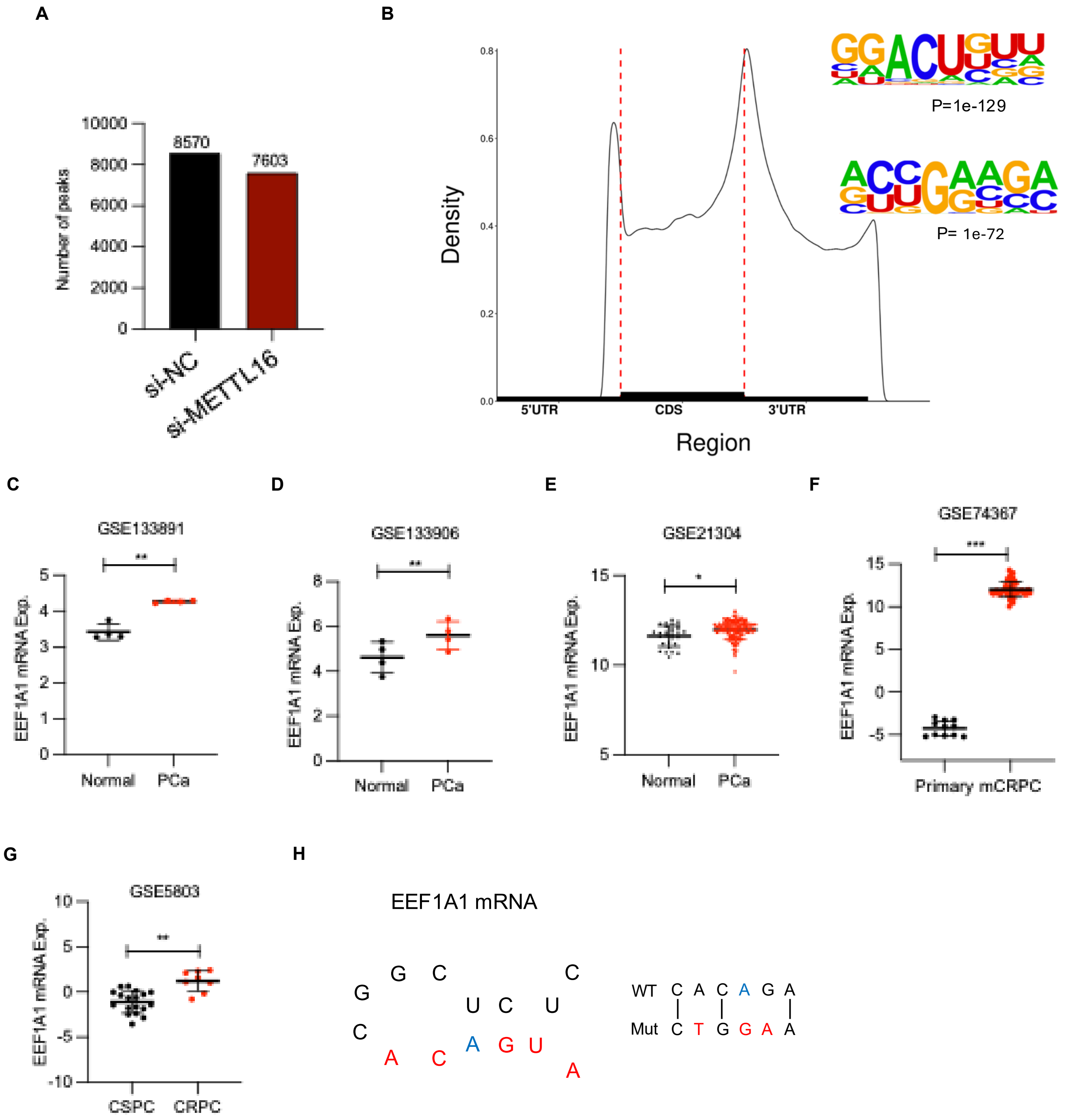

(A) m6A-seq of C4-2B showed a number of m6A peaks.; (B) The panel shows the normalized distribution of m6A peaks and identified m6A motif; (C-E) Relative mRNA expression of EEF1A1 in prostate cancer samples from GEO database; (F) Analysis of EEF1A1 expression in GSE74367 datasets; (G) Relative mRNA expression of METTL16 in CSPC and CRPC samples from GEO database;(H) Predicted secondary structures surrounding m6A peaks in EEF1A1 mRNA. *P< 0.05, **P<0.01, ***P<0.001.
